# Pattern of benzodiazepine use in psychiatric outpatients in Pakistan: a cross-sectional survey

**DOI:** 10.1186/1745-0179-5-9

**Published:** 2009-04-28

**Authors:** Syed Ahmer, Sumera Salamat, Rashid AM Khan, Saleem Perwaiz Iqbal, Imran Ijaz Haider, Ayesha Shabaz Khan, Mohsan Zafar

**Affiliations:** 1 Department of Psychiatry, Aga Khan University, Stadium Road, Karachi, Pakistan; 2 Department of Paediatrics and Child Health, Aga Khan University, Stadium Road, Karachi, Pakistan; 3 Fatima Memorial Hospital, Shadman, Lahore, Pakistan

## Abstract

**Background:**

Benzodiazepines (BDZ) are the largest-selling drug group in the world. The potential of dependence with BDZ has been known for almost three decades now. In countries like Pakistan where laws against unlicensed sale of BDZ are not implemented vigorously the risk of misuse of and dependence on these drugs is even higher. Previous studies have shown that BDZ prevalence among patients/visitors to general outpatient clinics in Pakistan may be as high as 30%. However, no research has been carried out on the prevalence of BDZ use in psychiatric patients in Pakistan.

**Methods:**

We carried out a cross-sectional survey over 3 months in psychiatry outpatient clinics of two tertiary care hospitals in Karachi and Lahore. Besides basic socio-demographic data the participants were asked if they were taking a BDZ at present and if yes, the frequency, route and dosage of the drug, who had initiated the drug and why it had been prescribed. We used chi-square test and t-test to find out which socio-demographic or clinical factors were associated with an increased risk of BDZ use. We used Logistic Regression to find out which variable(s) best predicted the increased likelihood of BDZ use.

**Results:**

Out of a total of 419 participants 187 (45%) of the participants had been currently using at least one BDZ. Seventy-three percent of the users had been using the drug for 4 weeks or longer and 87% were taking it every day. In 90% of cases the BDZ had been initiated by a doctor, who was a psychiatrist in 70% of the cases. Female gender, increasing age, living in Lahore, and having seen a psychiatrist before, were associated with an increased likelihood of using BDZ.

**Conclusion:**

The study shows how high BDZ use is in psychiatric outpatients in Pakistan. Most of the users were taking it for a duration and with a frequency which puts them at risk of becoming dependent on BDZ. In most of the cases it had been initiated by a doctor. Both patients and doctors need to be made aware of the risk of dependence associated with the use of BDZ.

## Background

After the discovery of Chlordiazepoxide in 1957 benzodiazepines (BDZ) soon became and still are the best-selling drugs in the world [1,2]. However, as early as in 1970s reports suggesting an association between long-term use of these drugs and tolerance started appearing in the medical literature. In 1981 Petrusson and Lader showed conclusively that a physiological withdrawal syndrome could develop with use of BDZ in doses considered to be in the therapeutic range [3]. The risk of dependence was estimated to be as high as 45% after 6 months of continuous use [3,4]. Since then concerns about dependence and withdrawal have prompted drug regulatory agencies of many countries to place restrictions on prescribing and over-the-counter sales of BDZ [5]. In many countries BDZ are licensed only for the short-term treatment of anxiety and insomnia, as a pre-anaesthetic and for alcohol detoxification [6]. In Pakistan while legally the BDZ can be purchased only after producing a doctor's prescription, at a practical level they can be very easily purchased without one.

Depending on the definition of BDZ use, period of observation and the population studied, estimates of BDZ use prevalence have ranged from as low as 0.2% to as high as 86% [7,8]. Generally, studies done on the general population report lower prevalence rates than those done in medically and psychiatrically ill population, and inpatient populations higher than outpatient populations [2,7-15].

A few studies have been conducted on the use of BDZ in Pakistan. The study by Raoof et al[16] showed that among patients and visitors at the outpatient clinics of a tertiary care hospital in Karachi 30.4% had used BDZ at some point in their life and 42% of the users had been using it for more than a year. Another study from Karachi showed 21.2% of inpatients at a tertiary care hospital were being prescribed BDZ[11] We did not come across any study exploring the pattern of BDZ use in psychiatric inpatient and outpatient populations in Pakistan.

In this study we wanted to find out the prevalence and pattern of BDZ use in psychiatric out patients in Pakistan. We also want to find out if there are any demographic or clinical variables that are associated with an increased likelihood of using BDZ in these populations, whose decision was it to start these medications, and why were they started in the first place.

## Methods

The study was conducted simultaneously in two tertiary care hospitals in Pakistan, the Aga Khan University Hospital in Karachi, and Fatima Memorial Hospital in Lahore. Both hospitals are private, fee-paying though the services at the latter are heavily subsidized and fees are much lower. Ethical approval for conducting the study was obtained from Ethical Review Committees of both the hospitals.

### Subjects

All patients attending the psychiatry outpatient clinics at both the centres for the first time (Initial patients) were eligible to be recruited in the study. There was no restriction on age as in Pakistan BDZ are sometimes used as antiepileptics, as well as on psychiatric diagnosis or any other variables. Only Initial patients were included as we did not wish the data to be influenced by the practice of consultant psychiatrists at the two centres.

### Data collection

Data was collected from May to July 2008. All consecutive patients attending the clinics during this period were invited to participate in the study. The data was collected from Monday to Friday, 9 am to 5 pm in Karachi, and Monday to Saturday 9 am to 2 pm in Lahore. As data was collected by one junior doctor each on both the sites because of the heavy volumes it was not possible to contact all the initial patients who attended the two clinics during this period. The number and details of those initial patients who could not be contacted, and details of those who refused to participate were not recorded.

The data was collected using a specially designed data collection form. The participants were initially asked for socio-demographic information like age, gender, marital status, education and occupation. They were then asked if they had ever seen a psychiatrist before their index consultation. The psychiatric diagnoses were recorded from the medical notes after the patients had been seen by the consultant psychiatrists at the participating institutions on their index (1^st^) visit.

The patients were then asked for the list of the medications they had been taking immediately prior to their index consultation and it was checked if they were taking any BDZ. Those who were found to be taking a BDZ were asked the duration, frequency, dose and route of drug use. They were asked who had first initiated the BDZ (as it is not uncommon in Pakistan for people to start taking a sleeping or anxiety pill themselves, or at the advice of a friend or a pharmacy salesman without any need for a prescription), and why the BDZ had been started initially. Diazepam equivalent doses were calculated using the Maudsley Prescribing Guidelines (page 199) [17]. The diazepam equivalent dose of bromazepam was not given in these guidelines or in Lexi-Comp's Drug Information Handbook so was taken from the benzo.org.uk website [18]

### Sample size requirements

Assuming the anticipated prevalence of benzodiazepine use at a tertiary care hospital as 30% and taking the precision of 5%, a minimum of 323 subjects were required, at 5% level of significance and 80% power.

### Data analysis

Analyses were done in SPSS 16.0. We used chi-squared test to analyse categorical variables like gender, city, whether they had ever seen a psychiatrist before the index presentation, etc, and t-test to analyse continuous variables like age. We performed Logistic Regression to find out which variable(s) best predicted the likelihood of someone taking a BDZ. We used BDZ taking as the dependent variable and the variables which were statistically significant or very close to statistical significance (age, gender, occupation, city, ever seen a psychiatrist before, and psychiatric diagnosis) as independent variables, entering all the variables at the same time. P value less than 0.05 was considered statistically significant.

## Results and discussion

### Analysis of pooled data

A total of 419 patients participated in the study, 225 in Karachi and 194 in Lahore. Median age of the participants was 30 years with an inter-quartile range of 24–42 years (range 5–86 years). Fifty-eight percent of the participants were male, 60% were married while 37% were single. Housewives (28%) were the largest occupational group, followed by unemployed (25%), skilled labourers (16%) and students (11%). Fifty eight percent of the participants had received at least secondary or higher education.

Sixty eight percent of participants had already seen a psychiatrist prior to being interviewed for this study. In terms of ICD-10 diagnosis about half (49.5%) of the participants were suffering from Mood (affective) disorders, followed by Schizophrenia, schizotypal and delusional disorders (23.6%), and Neurotic, stress-related and somatoform disorders (11.3%). About 7% of participants were not suffering from any psychiatric illness.

About 24% of the subjects, from Karachi reported to be suffering from cardiovascular diseases, including hypertension and ischemic heart disease. The proportion was found to be 6% among the subjects from Lahore. Diabetes mellitus was present among 10% of Karachi subjects and about 2% from Lahore. About 5% and 2% of subjects also reported to have gastro-intestinal problems from Karachi and Lahore, respectively (Data not shown).

Forty five percent (187/419) of participants reported that they were currently taking at least one BDZ. Of these, 176 patients were taking one, 11 were taking two and one was taking three BDZ. Frequency of the particular BDZ being taken was clonazepam 31.4%, lorazepam 23.9%, alprazolam 20.2%, bromazepam 14.9%, diazepam 9.6%, followed by midazolam, clobazam, and temazepam each taken by 0.5 to one percent of patients. The median diazepam equivalent dosage being taken was 7.49 milligrams with an inter-quartile range of 5–20 milligrams.

Participants had been taking the drug for a median duration of 12 weeks, with an inter-quartile range of 3–48 weeks. However, the range was very wide extending from 1 to 1440 weeks. Among users 73% (137/187) had been taking the drug for four or more weeks and 22% (41/187) (9.8% of the total sample) for more than one year. About 87% of the respondents were taking the BDZ daily, 3.7% about once a week, 5.9% less than once a week and 3.2% less than once a month. Almost all (98.4%) the patients were taking the drug orally with only one patient each taking it intramuscularly and intravenously.

In 70% of the cases the drug had been started by a psychiatrist while in 10% of cases the participants had started using the drug themselves. The drug had been initiated by a family physician in 8.6% of the cases, internist 6.4%, and other physicians and relatives in 0.5–2% of the cases. In 73% of the cases the drug was being prescribed/taken for insomnia, 12% relaxation, 10% anxiety and in 3.2% as antiepileptic.

The group taking BDZ was slightly older than the group which was not (mean age 35.8 vs 32.5 years, p = 0.02). The association between basic sociodemographic and clinical data, and the likelihood of taking benzodiazepines, is shown in Table 1. Occupation, city of residence, and the fact that a participant had previously seen a psychiatrist, were all statistically significantly associated with taking BDZ, while female gender and ICD-10 diagnosis were marginally statistically significant.

**Table 1:** Association between sociodemographic and clinical variables and the likelihood of taking benzodiazepines

Variable	Number (%)	Taking BDZ	P-value
Gender (n = 417)			0.058

Male	242 (57.9)	99 (40.9)	

Female	176 (42.1)	89 (50.6)	

			

Marital status(n = 419)			0.424

Married	249 (59.4)	116 (46.6)	

Unmarried	170 (40.6)	72 (42.4)	

			

Education (n = 419)			0.2

None	113 (27)	53 (46.9)	

Primary	65 (15.5)	34 (52.3)	

Secondary	81 (19.3)	37 (45.7)	

Intermediate	47 (11.2)	16 (34)	

Graduate	82 (19.6)	39 (47.6)	

Postgraduate	31 (7.4)	9 (29)	

			

**Occupation **(n = 418)			0.03*

Student	46 (11)	14 (30.4)	

Professional	31 (7.4)	10 (32.3)	

Housewife	118 (28.2)	66 (55.9)	

Retired	9 (2.2)	5 (55.6)	

Skilled labour	65 (15.6)	26 (40)	

Businessman	30 (7.2)	9 (30)	

Landlord	10 (2.4)	5 (50)	

Unemployed	108 (25.8)	53 (49.1)	

Unskilled labour	1 (0.2)	0 (0)	

			

City (n = 419)			0.004*

Karachi	225 (53.7)	86 (38.2)	

Lahore	194 (46.3)	102 (52.6)	

			

Ever seen psychiatrist (n = 419)			0.009*

Yes	284 (67.8)	140 (49.3)	

No	135 (32.2)	48 (35.6)	

			

ICD-10 Diagnosis (n = 390)			0.053

F00-09	7 (1.8)	5 (71.4)	

F10-19	19 (4.9)	14 (73.7)	

F20-29	92 (23.6)	36 (39.1)	

F30-39	193 (49.5)	90 (46.6)	

F40-48	44 (11.3)	17 (38.6)	

F50-59	2 (0.5)	0 (0)	

F60-69	2 (0.5)	0 (0)	

F70-79	2 (0.5)	0 (0)	

F80-89	1 (0.3)	1 (100)	

F90-98	1 (0.3)	0 (0)	

No psych illness	27 (6.9)	10 (37)	

* Statistically significant at the level of P < 0.05

• *n *is different for different variables as not all the participants had answered all the questions

The results of Simple Multiple Logistic Regression analysis are shown in Table 2. Age, gender and city of residence were the strongest predictors of the likelihood of someone taking a BDZ, while having previously seen a psychiatrist was at the margin of statistical significance.

**Table 2:** Factors predicting the likelihood of taking a BDZ.

Variable	Odds ratios* (95% C.I.)	P-value
Age	0.98 (0.97–0.99)	0.01*

Gender	0.61 (0.40–0.95)	0.03*

Occupation	0.99 (0.90–1.09)	0.85

City	0.51 (0.31–0.81)	0.005*

Ever seen psychiatrist	1.56 (0.97–2.49)	0.06

ICD-10 Diagnosis	1.01 (0.98–1.03)	0.65

Simple Multiple Logistic Regression Analysis

* Statistically significant at p < 0.05

### Differences between cities

We also analysed the data to see if there were differences between participants living in Karachi or Lahore in terms of their BDZ use. There was a statistically significant difference between Karachi and Lahore in the prevalence of psychiatric outpatients taking benzodiazepines, with more people in Lahore than in Karachi taking benzodiazepines (52.6% vs 38.2%, p = 0.004). There were gender-wise differences between cities in terms of BDZ use. In Karachi females were significantly more likely to take BDZ (F:M = 48% vs 31%, p = 0.01). However, no such difference was seen in Lahore (F:M = 54.5% vs 51.7%, p = 0.77). There was a huge difference between cities in terms of who had initiated the BDZ, in Lahore an overwhelming 95.1% of participants reported that the BDZ had been initiated by a psychiatrist, while in Karachi only 41.2% of participants had been initiated on BDZ by a psychiatrist. This difference was statistically significant (p < 0.001).

## Discussion

In this survey on point prevalence of BDZ use in people visiting psychiatry outpatient clinics for the first time in two cities of Pakistan, about half (45%) of respondents reported that they had been taking BDZ at the time of the interview. Among the users 72% had been taking the BDZ for 4 or more weeks and 87% had been taking it on a daily basis.

Direct comparison of this prevalence rate with other studies is made slightly difficult by different study criteria used by different studies, particularly the definition of BDZ use, population studied, and duration over which BDZ use was measured[7] There were two previous studies from Pakistan which had assessed BDZ prevalence. In the study by Raoof et al in which the sample was outpatients and visitors in general (non-psychiatric) outpatient clinics at a tertiary care hospital, and which collected data on lifetime prevalence, the prevalence was 30.4%[16] In the only other study from Pakistan on this topic which looked at the proportion of both non-psychiatric and psychiatric inpatients who had been prescribed BDZ during their hospital stay the point prevalence of BDZ prescription was 21.2%[11]

Studies from other parts of the world show that prevalence rates vary hugely ranging from 7.5% *currently taking *a BDZ in French general population[15], 9.6% past month use in Lebanese general population[19], 19.1% taking BDZ regularly (one or more doses per week) at the time of admission to a Sydney teaching hospital[10], 33% taking BDZ *at the time of admission *to an Internal Medicine unit in France[12], 41.4% prescribed BDZ to medical/surgical inpatients in USA[9], and 86% *lifetime prevalence *of BDZ use in patients admitted to a Swiss psychiatric hospital[8]. The 45% point prevalence of BDZ use in psychiatric outpatients found in our study seems to be higher than the prevalence found in previous studies from Pakistan, and closer to the prevalence in inpatient units in international studies.

Putting the three facts together that the drug had been initiated by a psychiatrist in 70% of the cases and doctors in general in 89% of the cases, that 72% of users were taking it for 4 or more weeks, and that 87% were taking it on a daily basis, highlights the iatrogenic origin of this major public health concern. Either the doctors are not educating patients about the maximum duration of how long BDZ can be safely taken for, or the somehow the patients are not receiving this message. Some research suggests that the former hypothesis may be more accurate. A survey of general practitioners in Thailand showed that about 23% of general practitioners agreed that BDZ can be prescribed regularly for more than 1 month and about 6% thought that they could be prescribed for more than 4 months to 6 months[20] On the other hand, there is some research evidence which suggests that patients tend to use less BDZ medications than prescribed by their doctors which refutes the second hypothesis[21]

The three variables which were significantly associated on logistic regression with the likelihood of taking BDZ were female gender, age and the city of residence. The finding of an increased prevalence of BDZ use in females is very consistent across studies and countries[7,15,19,22] One possible explanation may be the higher prevalence of mood and anxiety disorders in females as these two groups of disorders accounted for over two thirds of all patients in our study. Age has also been associated with increasing risk of BDZ use in other studies[15,19]. However, the group taking BDZ in our study was only slightly older that the group not taking BDZ (mean 35.8 vs 32.5 years) and the Odds Ratio 0.98 (95% C.I. 0.97–0.99) was very close to 1, it is difficult to interpret this finding.

There were several statistically significant differences between respondents in Karachi and Lahore in terms of prevalence of BDZ use, difference between proportion of females taking BDZ, and whether a psychiatrist had or had not initiated the BDZ. In the absence of any previous research in this area this needs to be studied more before it can be interpreted meaningfully.

Though we inquired about the initiation of BDZ, we did not assess as why the drug consumption continued, and this can be regarded as a limitation of the study. We feel that there may have been a communication gap either from the side of the psychiatrist (might or might not have advised BDZ continuation), or from the side of the patient as he/she may not have complied with the psychiatrist's advice.

## Conclusion

In this study about half of the population studied had been taking BDZ at the time of the interview, 72% of the users had been taking the drug for than 4 or more weeks, 87% of users were taking it daily and in 90% of the cases the BDZ had been initiated by a doctor. These finding highlight the scale of this public health problem. In spite of availability of modern dugs and psycho-therapeutic measures, it shall not be easy to contain the subsequent problems and complications, as these measures would inculcate some direct and indirect costs. Education, at regular intervals, may assist in controlling this issue remarkably.

The data suggests that the primary prevention of BDZ misuse must start with education of doctors in general and psychiatrists in particular that they should educate every patient they prescribe BDZ to about the potential of addiction with these drugs.

## List of abbreviations

BDZ: Benzodiazepine(s).

## Competing interests

None of the authors have any competing interests to declare. No funding was obtained for carrying out this research.

## Authors' contributions

SA conceived the idea. SA, RK and SI developed the protocol. SS, RK, IH, AK and MZ collected the data. SS carried out the data entry. SA and SI carried out the analyses. All authors reviewed the manuscript critically and approved it.
